# Effect of Weight-Bearing in Conservative and Operative Management of Fractures of the Base of the Fifth Metatarsal Bone

**DOI:** 10.1155/2017/1397252

**Published:** 2017-12-26

**Authors:** Jae-Yong Park, Hyong-Nyun Kim, Yoon-Suk Hyun, Jun-Sik Park, Hwan-Jin Kwon, Sae-Hyun Kang, Gab-Lae Kim

**Affiliations:** ^1^Department of Orthopaedic Surgery, Hallym University Sacred Heart Hospital, Hallym University College of Medicine, Anyang, Republic of Korea; ^2^Department of Orthopaedic Surgery, Kangnam Sacred Heart Hospital, Hallym University College of Medicine, Seoul, Republic of Korea; ^3^Department of Orthopaedic Surgery, Kangdong Sacred Heart Hospital, Hallym University College of Medicine, Seoul, Republic of Korea

## Abstract

**Background:**

There is no established principle regarding weight-bearing in conservative and operative management of fifth metatarsal base fractures.

**Methods:**

We reviewed 86 patients with acute fifth metatarsal base fractures. Conservatively treated late or early weight-bearing patients were assigned to Group A or C, respectively. Operatively treated late or early weight-bearing patients were assigned to Group B or D, respectively. Results were evaluated by clinical union, bone resorption, and the American Orthopaedic Foot and Ankle Society (AOFAS) and Visual Analogue Scale (VAS) scores.

**Results:**

All 4 groups had bone union at a mean of 6.9 weeks (range, 5.1–15.0). There were no differences between the groups in the AOFAS and VAS scores. In the early weight-bearing groups, there were fewer cases of bone resorption, and the bone unions periods were earlier.

**Conclusions:**

Early weight-bearing may help this patient population. Moreover, conservative treatment could be an option in patients with underlying diseases.

## 1. Introduction

**Figure 1:**
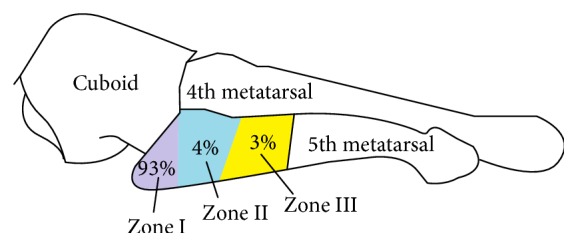
Classification in Zones (Dameron, Lawrence, and Quill) drawn by K. Han, MD.

The metatarsal bone fracture (International Classification of Diseases, Tenth Edition [ICD-10] code S92.3) accounts for 35% of all foot fractures [1]. Most of these fractures are observed in the fifth metatarsal bone followed by the third, second, first, and fourth metatarsal bones [2]. Jones [3] first reported the fracture of the base of the fifth metatarsal bone in 1902. Dameron Jr. and Quill Jr. [4, 5] classified the proximal portion of the fifth metatarsal fracture as the zone injury. According to this classification, zone 1 fractures are tuberosity avulsion fractures, with an incidence of 93%. Zone 2 fractures (4%) are metadiaphyseal fractures called Jones' fractures. Zone 3 fractures are proximal shaft stress fractures (3%) [6] (Figure 1). An indirect force usually causes the fifth proximal metatarsal fractures. Among them, zone 3 fractures are usually due to repetitive trauma, unlike zones 1 and 2 fractures.

Although more conservative treatment for zone 1 fractures and more surgical treatment for zone 3 fractures are the trend in treating proximal fifth metatarsal fractures, there is no clear determination of which management is superior. Moreover, there is no established principle in use and load of weight-bearing and period or method of immobilization for conservative treatment [7–12]. Therefore, this study aimed to compare the outcomes of conservative and operative treatment and to evaluate the effect of early weight-bearing in fifth metatarsal base fractures, except for stress fractures.

## 2. Patients and Methods

### 2.1. Patients

Eighty-six patients with a fracture of the base of the fifth metatarsal bone who underwent 6 months of follow-up observation participated in this study. Patients received treatment in either the hospital or outpatient clinic from March 2010 to August 2012 sequentially. We performed this study retrospectively and excluded patients with accompanying injuries and stress fractures. An accompanying injury includes other bone fractures in the ipsilateral foot and another injured area that interrupts weight-bearing. When patients had previous symptoms in the fifth metatarsal base and lateral cortical thickening seen on a plain radiograph, we determined that they had a stress fracture. Forty-four subjects received cast immobilization for conservative treatment, and 42 subjects received operative treatment. Overall, 46 were trained for full weight-bearing 3 days after cast immobilization, and 40 had limited weight-bearing for up to 6 weeks.

We separated these patients into four groups by weight-bearing onset and treatment options. Among patients treated conservatively, 20 who performed late weight-bearing were Group A and 24 who performed early weight-bearing were Group C. Among patients treated operatively, 20 who performed late weight-bearing were Group B and 22 who performed early weight-bearing were Group D (Table 1). The protocol used to perform a retrospective review of patient records was approved by the Institutional Review Board of Kangdong Sacred Heart Hospital (no. 14-2-08).

### 2.2. Treatment Methods

**Figure 2:**
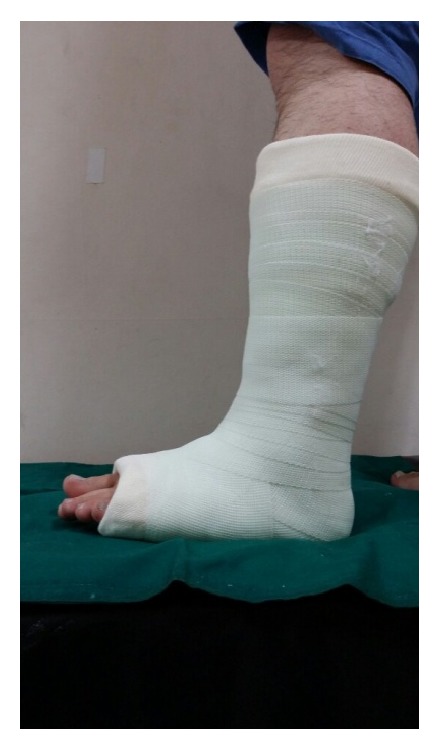
Short-leg cast (lateral).

**Figure 3:**
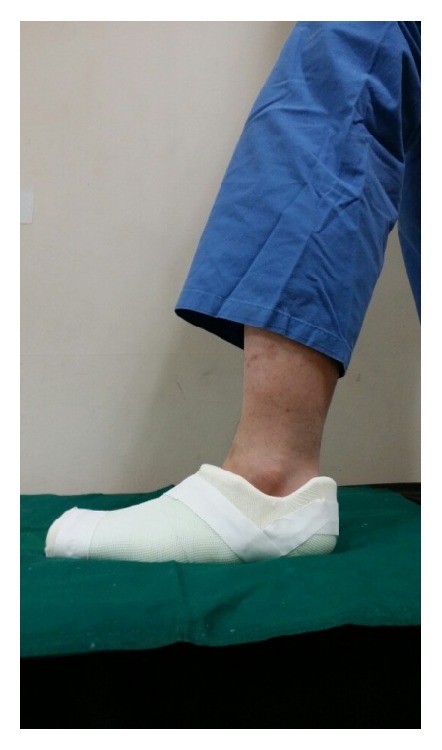
Foot cast (lateral).

The early weight-bearing groups were trained to use full weight-bearing 3 days after cast immobilization, whereas the late weight-bearing groups were not permitted to use full weight-bearing for 6 weeks after surgery (they were educated to stand on toe tip with crutch). For immobilization, we used the short-leg cast (28 patients) in the early stage of the study (March 2010 to December 2010) (Figure 2). In the later stage of the treatment, we used the foot cast (58 patients) (January 2011 to August 2012) (Figure 3). All patients had short-leg splint from trauma to cast application.

We performed surgical fixation in patients with displaced fractures (≥2 mm in the foot in the oblique view). The operations were performed under either general or spinal anesthesia. Patients lay in supine position, and a tourniquet was used in the proximal part of the thigh. Fixation materials were a screw (65.9%, 29 patients) or tension band wiring (29.5%, 13 patients). Two patients received an auto-bone graft from the calcaneus for their bone defects. H. N. K. followed the late weight-bearing protocol (Group B). G. L. K. followed the early weight-bearing protocol (Group D).

### 2.3. Evaluation Methods

We evaluated clinical bone union in all groups. Clinical union was defined as plain film radiographic evidence of bone healing and minimal to no pain clinically [13]. We also tried to check the radiological bony union time, but the data of radiological bony union time is not suitable. This was because our surgical method is for primary bone union. We also evaluated bone resorption using plain radiography. The function status was assessed using the American Orthopaedic Foot Ankle Society (AOFAS) Lesser Metatarsophalangeal-Interphalangeal Scale and the pain Visual Analogue Scale (VAS) [14, 15]. We also assessed complications posttreatment.

All statistical analyses were performed using Statistical Package for the Social Sciences version 22.0 (IBM Corp.). Descriptive statistics were calculated for each parameter and consisted of the mean, standard deviation (SD), 95% confidence interval (CI), and range. The result of each group was compared using the independent *t*-test. Results were considered significant when *p* value < 0.05.

## 3. Results

There were 38 men and 48 women with age ranging from 11.0 to 91.1 years (mean 42.79 ± 18.8 years, 95% CI 38.75 to 46.82). Demographic data compared among the 4 groups were not different (Table 2). All groups had more female patients than male patients, but Group B had 2 more male patients than female patients. Most patients had zone 1 fracture (89.5%, 77 patients), and the others had zone 2 fractures (10.5%, 9 patients). Some patients had diabetes mellitus (ICD-10 code E10-14) and osteoporosis (ICD-10 code M81). A misstep was the main cause of the fracture, accounting for 68 patients followed by a traffic crash (pedestrian; 13 patients) and direct injury (5 patients) (Table 3).

Treatment was initiated at an average of 0.51 days (range 0–3 days, SD .72) after the fracture occurred. Cast immobilization in the conservative treatment groups was started at an average of 2.93 days (range 1–5 days, SD 1.11) from the injury. Group A began cast immobilization at an average of 3.10 days (range 1–5 days, SD 1.25), whereas Group C began cast immobilization at an average of 2.8 days (range 1–5 days, SD .98) from the injury, which was not significantly different. Group B began cast immobilization at an average of 8.10 days (range 5–15 days, SD 2.36), whereas Group D began cast immobilization at an average of 5.81 days (range 2–16 days, SD 3.02) from the injury, which was significantly different. This was probably because there was some difference in the cast protocol between Groups B and D. Group D received cast immobilization as soon as the operation had finished for early weight-bearing, but Group B received cast immobilization after edema had completely resolved postoperatively. The total cast periods were an average of 4.0 weeks (range 3.5–5.0 weeks, SD .25) in the conservative treatment group and 6.1 weeks (range 6.0–8.0 weeks, SD .31) in the operative treatment group. A crutch was used for 6.3 weeks (range 6.0–7.0 weeks, SD .38), 6.9 weeks (range 6.5–7.0 weeks, SD .21), 0.8 weeks (range 0.6–1.0 week, SD .12), and 1.1 weeks (range 1.0–2.0 weeks, SD .27) in Groups A, B, C, and D, respectively. We performed the operations at an average of 3.8 days (range 1.0–8.0 days, SD 1.74) from injury in the late weight-bearing groups and 4.3 days (range 1.0–9.0 days, SD 2.21) in the early weight-bearing groups, which was not significantly different.

**Figure 4:**
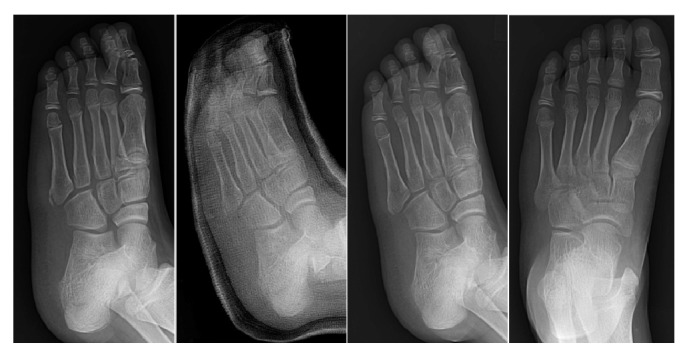
Nonoperative treated, early weight-bearing patient's radiograph. 12-year-old male; radiograph of pre-cast application and 1-, 3-, and 6-month follow-up (from left to right).

**Figure 5:**
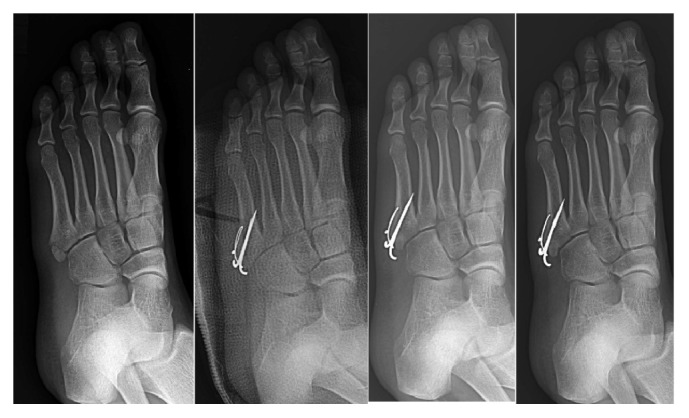
Operative treated, early weight-bearing patient's radiograph. 24-year-old female; radiograph of preoperation and 1-, 3-, and 6-month follow-up (from left to right).

**Figure 6:**
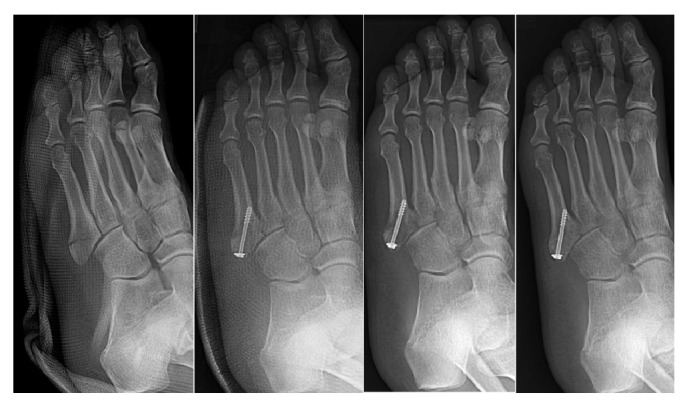
Operative treated, late weight-bearing patient's radiograph. 30-year-old male; radiograph of preoperation and 1-, 3-, and 6-month follow-up (from left to right).

Clinical bony union was confirmed at an average of 6.9 weeks (range 5.1–15.0 weeks, SD 1.48) in all patients (Figures 4–6). Two subjects who underwent conservative treatment had delayed union (a clinical union time more than 3 months), but all subjects had union at 6 months of observation. Patients with late union had diabetes and osteoporosis. Each patient was in the early and late weight-bearing groups. Clinical bone union was confirmed at an average of 7.9 weeks (range 6.2–15.0 weeks, SD 1.95), 6.9 weeks (range 6.1–8.0 weeks, SD .55), 6.8 weeks (range 5.5–14.3 weeks, SD 1.70), and 6.1 weeks (range 5.1–7.3 weeks, SD .57) in Groups A, B, C, and D, respectively. The late weight-bearing groups (Groups A and B) had clinical bone union at an average of 7.4 weeks (SD 1.50), whereas the early weight-bearing groups had clinical bone union at an average of only 6.5 weeks (SD 1.33), which was significantly different.

Bone resorption in the early stage was found in 4% (Group C, 1 patient) and 9% (Group D, 2 patients) of patients in the early weight-bearing groups. However, it was more frequent in the late weight-bearing groups (Group A: 25%, 5 patients; Group B: 20%, 4 patients). However, the significance was not calculated because of the small number.

The AOFAS and VAS scores were evaluated at 1, 3, and 6 months, but there was no significant difference among the groups (Table 4). Moreover, there were no significant complications, such as infection, nonunion, and malunion.

## 4. Discussion

Since Sir Jones first reported the fracture of the base of the fifth metatarsal bone in 1902, the fracture was named the Jones fracture [3]. He reported the outcome of conservative treatment of four fracture cases, and the report brought lots of different opinions and arguments on the treatment of fractures of the base of the fifth metatarsal bone. However, there has been no definite principle for treatment during the non-weight-bearing, that is, a period of cast immobilization or use of orthoses. Fixation using a metal wire, cannulated screw, or tension band wire is used for operative treatment, but there have been several controversies regarding their advantage and disadvantage and the period of weight-bearing.

Regarding the effect of weight-bearing, Torg et al. [16] reported the results of the conservative and operative treatment of 46 patients with fractures of the base of the fifth metatarsal bone. Among 25 patients with an acute fracture, 15 had treatment composed of 6–9 weeks of non-weight-bearing and a short-leg cast. The others (10 patients) had weight-bearing with an orthosis or cast. In the non-weight-bearing group, 14 patients had bone union at an average of 7 weeks. However, in the weight-bearing group, only 4 patients achieved bone union; this questioned the use of early weight-bearing in nonoperative treatment. However, Choi et al. [9] reported that they observed bone union in all 58 subjects who received nonoperative treatment with early full weight-bearing at an average of 45.5 days. Subjects had a fracture of the base (zones I and II) of the fifth metatarsal bone, and full weight-bearing was allowed right after the injury with 4 weeks of casting.

Regarding fixation, Pietropaoli et al. [8], in their biodynamic study of Jones fractures, reported that operative treatment using screw fixation was more effective than conservative treatment to prevent bone movement reduction from fixation because of its pull-out strength. Besides, Suh et al. [7] reported early clinical bone union (mean 4.81 weeks) using full weight-bearing with a tolerable range after a mean of 3.9 weeks of partial weight-bearing in the zone 1 or 2 operational treatment group (cannulated screw or tension band wiring).

In our study, 86 subjects were classified into either an operative or nonoperative treatment group and then further into two groups: the early weight-bearing or late weight-bearing group. There was no significant difference in pain or bone resorption between the operative and nonoperative treatment groups. Overall, 22.5% of subjects in the late weight-bearing group had bone resorption, which was higher by 6.5% compared to that in the early weight-bearing group. Bone union was achieved 1 week later in the late weight-bearing group than in the early weight-bearing group. Thus, early weight-bearing was considered to prevent bone resorption and perhaps improve bone union. Therefore, it was considered better to use weight-bearing in the early stage within the allowable range of pain experienced by patients.

From January 2011, we used the foot cast instead of the short-leg cast. Patients were usually satisfied with the foot cast in terms of lightness and breathability. This cast could not fully restrict the ankle motion, but it could restrict ankle inversion and eversion well. Thus, influence of the peroneus brevis was not a factor in our study.

There are some limitations of this study. First, the sample size was small, so it was difficult to identify a statistically significant difference between each group's measurement; additionally, sensitivity analysis for the potential influence of an unmeasured variable on outcome was lacking. Second, two different surgeons treated each group (Groups B and D); this means that we had no pure comparison group, and our result may have been influenced by the technical difference between the surgeons. Third, we had no objective standard of a bone resorption state, so the result may not be reasonable. Finally, this article is based on a retrospective study, so its results may need to be proven by a further prospective study.

## 5. Conclusions

All subjects with an acute fracture of the base of the fifth metatarsal bone who received either operative or conservative treatment had bone union, but the early weight-bearing groups showed faster bone union and less bone resorption. The VAS and AOFAS scores were not significantly different at the final observation. The early weight-bearing training was considered to shorten the period of bone union. Therefore, nonoperative treatment with weight-bearing could be considered an effective treatment for patients with underlying diseases.

## Figures and Tables

**Table 1:** Patient separation.

	Late weight-bearing (40)		Early weight-bearing (46)	
	Conservative Tx (20) 〈Group A〉 23.3%	Operative Tx (20) 〈Group B〉 23.3%	Conservative Tx (24) 〈Group C〉 27.9%	Operative Tx (22) 〈Group D〉 25.6%
Mean age/male/female	44.2/8/12	41.3/11/9	38.8/10/14	47.2/9/13
Treatment initiation (days)	0.8	0.5	0.4	0.4
Operation (days)	-	3.8	-	4.3
Cast apply (days)	3.1	8.1	2.8	5.8
Cast off (weeks)	4.1	6.0	3.9	6.1
Crutch use (weeks)	6.3	6.9	0.8	1.1

**Table 2:** Demographic data.

	Late weight-bearing (40)		Early weight-bearing (46)	
	Conservative Tx (20) 〈Group A〉 23.3%	Operative Tx (20) 〈Group B〉 23.3%	Conservative Tx (24) 〈Group C〉 27.9%	Operative Tx (22) 〈Group D〉 25.6%
Mean age (years)	44.2 (11.0–91.1)	41.3 (16.3–81.2)	38.8 (12.1–77.9)	47.2 (17.2–88.8)
Sex (male : female ratio)	40 : 60	55 : 45	41.7 : 58.3	40.9 : 59.1
Fracture type (case)	Zone I—18 (90%)	Zone I—17 (85%)	Zone I—21 (87.5%)	Zone I—21 (95.5%)
	Zone II—2 (10%)	Zone II—3 (15%)	Zone II—3 (12.5%)	Zone II—1 (4.5%)
^*∗*^DM (case)	4 (20%)	2 (10%)	3 (12.5%)	4 (18.2%)
Osteoporosis (case)	3 (15%)	3 (15%)	3 (12.5%)	1 (4.5%)

^*∗*^DM: diabetes mellitus.

**Table 3:** Modes.

Mode	Misstep	Traffic accident	Direct injury
	68 cases (79.0%)	13 cases (15.1%)	5 cases (5.8%)

**Table 4:** Results.

	Late weight-bearing (40)		Early weight-bearing (46)	
	Conservative Tx (20) 〈Group A〉 23.3%	Operative Tx (20) 〈Group B〉 23.3%	Conservative Tx (24) 〈Group C〉 27.9%	Operative Tx (22) 〈Group D〉 25.6%
Clinical union (weeks)	7.9 (6.2–15.0)	6.9 (6.1–8.0)	6.8 (5.5–14.3)	6.1 (5.1–7.3)
Bone resorption (cases)	5 (25%)	4 (20%)	1 (4%)	2 (9%)
AOFAS^*∗*^ score (1/3/6 months)	77.1/88.4/94.7	72.2/82.3/96.6	73.4/84.2/97.8	75.2/89.9/99.1
VAS^*∗∗*^ score (1/3/6 months)	2.5/1.9/0.3	3.7/2.3/0.3	3.3/1.2/0.6	2.9/1.8/0
Complications	1 delayed union	-	1 delayed union	-

^*∗*^AOFAS: American Orthopaedic Foot Ankle Society; ^*∗∗*^VAS: Visual Analogue Scale.
